# A multi-modal sensing system for human-robot interaction through tactile and proximity data

**DOI:** 10.3389/frobt.2025.1581154

**Published:** 2025-06-10

**Authors:** Gianluca Laudante, Michele Mirto, Olga Pennacchio, Salvatore Pirozzi

**Affiliations:** Engineering Department, University of Campania “Luigi Vanvitelli”, Aversa, Italy

**Keywords:** human-robot interaction, human-robot collaboration, tactile sensor, proximity sensor, multi-modal, modular

## Abstract

**Introduction:**

The rapid advancement of collaborative robotics has driven significant interest in Human-Robot Interaction (HRI), particularly in scenarios where robots work alongside humans. This paper considers tasks where a human operator teaches the robot an operation that is then performed autonomously.

**Methods:**

A multi-modal approach employing tactile fingers and proximity sensors is proposed, where tactile fingers serve as an interface, while proximity sensors enable end-effector movements through contactless interactions and collision avoidance algorithms. In addition, the system is modular to make it adaptable to different tasks.

**Results:**

Demonstrative tests show the effectiveness of the proposed system and algorithms. The results illustrate how the tactile and proximity sensors can be used separately or in a combined way to achieve human-robot collaboration.

**Discussion:**

The paper demonstrates the use of the proposed system for tasks involving the manipulation of electrical wires. Further studies will investigate how it behaves with object of different shapes and in more complex tasks.

## 1 Introduction

In the rapidly evolving landscape of robotics, the collaboration between humans and robots has emerged as a pivotal research domain, marked by its potential to redefine the way we interact with robotic systems. One fundamental aspect of this collaboration involves the integration of human guidance mechanisms based on multi-modal sensing systems, empowering robots to learn and perform tasks through human interaction.

Thanks to the development of collaborative robots, known as “cobots”, many researchers started to investigate innovative solutions and methodologies for human-robot interaction, also inspired by typical collaboration techniques among humans. Madan et al. (2015) studied haptic interaction patterns that are typically encountered during human-human cooperation, with the aim to simplify the transfer of a collaborative task in a human-robot context. Kronander and Billard (2014), considering the Learning from Demonstration framework, present interfaces that allow a human teacher to indicate compliance variations by physically interacting with the robot during task execution. Singh et al. (2020) propose a collaborative dual-arm teleoperation setup, where one of the two arms acts as a controller and the other as a worker. The authors exploit the possibility of using joint torque commands for controlling the robotic arms and the integrated torque sensors at each joint actuator to transfer the motion from the controller to the worker, and the external forces sensed by the worker back to the controller. Another example of HRI is presented in (Grella et al., 2022), where authors exploit contact information provided by distributed tactile sensors, acting as the physical communication interface between the control system and the human, to ensure the robot moves only when the operator intentionally decides to move it. In this scenario, human safety is an important element as it contributes to efficient cooperation with the collaborative robots in several application fields (Zacharaki et al., 2020; Chandrasekaran and Conrad, 2015). Moreover, the human factor is also the focus of the Industry 5.0. In particular, the human behaviour modeling in industrial HRI manufacturing is analyzed in (Jahanmahin et al., 2022). These works represent, certainly not exhaustively, examples that demonstrate the interest in realizing easy and safe collaboration among humans and robots. It is worth noticing that in all of these examples there is a device specifically added to the system whose only purpose is to serve as an interface between the human operator and the robot.

Based on the complexity of the task that robots are requested to perform and the unstructured environments where some of these tasks are carried out, robotic systems are equipped with several sensing systems also depending on the type of interaction needed. Among these, tactile and proximity sensors play an increasingly interesting role. By reporting only some recent application examples, tactile sensors can be used for detecting the directionality of an external stimulus (Gutierrez and Santos, 2020), for grasp stabilization, by predicting possible object slippage (Veiga et al., 2018), or for haptic exploration of unknown objects, by extracting information such as friction, center of mass, inertia, to exploit for robust in-hand manipulation (Solak and Jamone, 2023). A wide list of the touch technologies involved in the interaction has been recently explored in (Olugbade et al., 2023). Proximity sensors can be used for implementing safety algorithms like obstacle detection and/or avoidance. In (Cichosz and Gurocak, 2022), the authors use ultrasonic proximity sensors mounted on a robotic arm for achieving collision avoidance in a pick-and-place task with the presence of a human operator in the same workcell. Other examples are detailed in (Moon et al., 2021; Liu et al., 2023), where capacitive proximity sensors are exploited for real-time planning of safe trajectories considering obstacles.

Clarified that the HRI research field is wide and intricate from both technology and methodology points of view, this paper focuses on the development of a modular sensing system that can be used as an interface in collaboration tasks, where humans guide robots in executing specific operations, allowing a learning process that aims to autonomous execution, and also for not collaborative tasks where sensing is required. Recently, the combination of different sensors is increasingly used, through sensor fusion or machine learning techniques, to tackle more complex tasks. To give some examples, in (Govoni et al., 2023) tactile sensors and proximity sensors are both used in a wiring harness manipulation task, while (Strese et al., 2017) classifies the texture of objects by considering information such as images, audio, friction forces, and acceleration. Fonseca et al. (2023) propose a flexible sensor based on piezoresistive and self-capacitance technology that can be applied to the robot links and used for hand guidance or collision avoidance. Similarly, Yim et al. (2024) present a sensor integrating electromechanical and infrared Time-of-Flight technologies to enhance safety during physical Human-Robot Interactions. Also, especially when dealing with non-rigid objects, it is not uncommon to fuse heterogeneous data coming from multiple sensors (Nadon et al., 2018).

Following this direction, this paper proposes a multi-modal sensing system, constituted by a modular solution, which can integrate different combinations of tactile and proximity sensors, together with a suitable methodology for exploiting these sensors during the execution of human-robot interaction tasks. From technology point of view, the design has been optimized based on previous experiences of some of the authors on tactile sensors (Cirillo et al., 2021a) and proximity sensors (Cirillo et al., 2021b). From a methodology point of view, for human-robot collaboration, the tactile fingers can be used, by means of specifically defined indicators, as the interface for controlling the robot motion during the teaching phase, avoiding the addition of specific tools in the setup. Instead, the proximity sensors are exploited for implementing collision avoidance and/or a method for moving the end effector in a contactless fashion. Differently from similar systems, the proposed solution does not require the addition of a sensing skin to the robot (like in Fonseca et al. (2023)) or a specific tool integrating the sensors (like in Yim et al. (2024)) since all the sensing components are already included in the modular end effector. The developed sensing system allows an extension of preliminary results presented in Laudante and Pirozzi (2023), increasing the degrees-of-freedom (DOFs) available for the robot guidance, covering the 3-D space and not only a plane. Additionally, the proposed modular solution presents a common mechanical base designed to guarantee easy integration in commercial parallel grippers. Some demonstrative tests are reported to show the validity of the proposed methodology.

The rest of the article is organized as follows. Material and methods Section 2 presents first the proposed tools, detailing the characteristics of the embedded tactile sensors in Section 2.1 and proximity sensors in Section 2.2, and then the developed methodology to exploit the sensors for Human-Robot Interaction in Section 2.3. Section 3 reports demonstrations to show the effectiveness of the presented technologies and methodology. Finally, Section 4 concludes the article by discussing on possible future developments.

## 2 Materials and methods

### 2.1 Tactile finger

**FIGURE 1:**
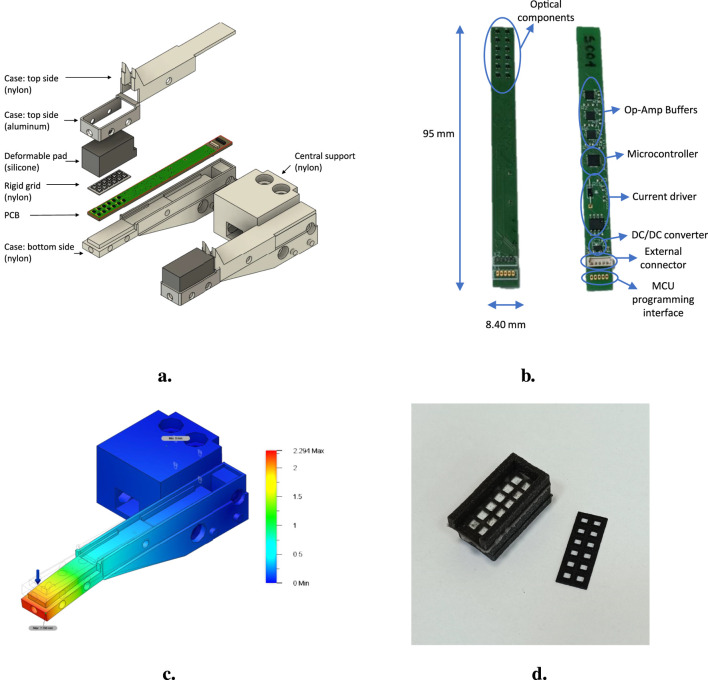
**(a)** CAD of the tactile finger showing the mechanical components. **(b)** Electronic board of the tactile finger. **(c)** FEA results for the tactile finger. **(d)** Samples of deformable pad and rigid grid.

The designed finger contains a tactile sensor based on optoelectronic technology and is a modified version of the finger previously presented in (Cirillo et al., 2021a). The following text briefly describes the sensing technology and the designed mechanical components, which can be seen in the pictures in Figure 1a.

#### 2.1.1 Sensing technology

The tactile sensor is based on the sensing technology reported in (Cirillo et al., 2021a) but with a smaller form factor. The sensor has 12 sensing points, here called “taxels”, each one constituted by a photo-reflector (NJL5908AR by New Japan Radio) that is the combination of a Light Emitting Diode (LED) and a phototransistor optically matched. The core part of the sensor is a Printed Circuit Board (PCB), where the 12 photo-reflectors are organized in six rows and two columns with a spatial resolution equal to 3.55 mm. The LEDs are driven by an adjustable current source (LM334 by Texas Instrument), while low-power operational amplifiers (ADA4691 by Analog Devices) act as a buffering stage, decoupling the outputs of the phototransistors and the inputs to the Analog-to-Digital converters (ADCs). Finally, a low-power microcontroller (PIC16F19155 by Microchip) acquires the phototransistor signals through 12 low-noise ADCs with 12− bit resolution. Figure 1b shows the manufactured PCB that highlights the abovementioned components. Table 1, instead, reports some characteristics of the tactile sensor that have been evaluated exeperimentally as reported in Cirillo et al. (2021a).

**TABLE 1:** Characteristics of the tactile sensor.

Number of photo-reflector	12
Sensing area	22.6×8.4 ${mm}^{2}$
Spatial resolution	3.55 mm
Sampling frequency	500 Hz
Response time	<0.01 s
Hysteresis error	≈5 %
Repeatability error	≈3 %
Sensitivity	0.018 V/N

The photo-reflectors are positioned underneath a deformable pad, which transduces deformations into contact information. In fact, the light emitted by each LED is reflected by the bottom part of the deformable pad and reaches the corresponding phototransistor in an amount dependent on the local deformation of the pad. Voltage signals from the sensor can be obtained by interrogating the microcontroller via serial interface, and this is done using a ROS (Robot Operating System) node running on a computer connected to the sensor via USB.

#### 2.1.2 Mechanical components

All the mechanical parts constituting the finger are shown in Figure 1a: the deformable pad, a rigid grid, and a case divided into three pieces. These parts are described in the following.

The deformable pad is made of silicone (PRO-LASTIX by Prochima) with a hardness equal to 20 Shore A due to its good elastic properties and low hysteresis. The pad presents cells with a parallelepiped shape on the bottom face (see Figure 1d) which are aligned with the optoelectronic components. The specific shape of these cells has been selected after an optimization design process as reported in Laudante et al. (2023). The whole pad is black, while the ceiling of each cell is white. In this way, it is possible to achieve good reflection of the light of the LEDs and to avoid interferences coming from near taxels or external light sources.

To ensure perfect alignment between the cells in the pad and the sensing points onto the PCB, a rigid grid with protruding parts that fit into the cells is glued to the electronic board. Additionally, the thickness of the grid is such that it guarantees that the reflective surface, i.e., the ceiling of the cells, cannot reach a distance less than 0.5 mm from the photo-reflectors, avoiding a non-monotonic behavior of these components. A sample of the rigid grid is shown in Figure 1d.

Finally, the PCB described in the previous section is housed in a case consisting of three pieces. The one indicated as “bottom side” in Figure 1a, in addition to keeping the PCB in position, gives rigidity to the finger. In particular, during the design of this part, Finite Elements Analysis (FEA) has been exploited to select appropriate dimensions to obtain a robust mechanical component considering the maximum force applicable on the sensor pad before signal saturation, equal to 40 N (evaluated experimentally). The results of this analysis are shown in Figure 1c. The top side of the case consists of two parts, one to cover the PCB and the other to enclose and lock the silicone pad.

The rigid grid, the bottom side of the case and the top side part which cover the PCB are made of nylon PA12 and are 3D printed. The thickness of the layers is 60  μ m and the precision of the 3D printer used is ±0.30 mm. The second part of the top side of the case, instead, is realized in aluminum with CNC machining and a precision of ±0.1 mm.

**FIGURE 2:**
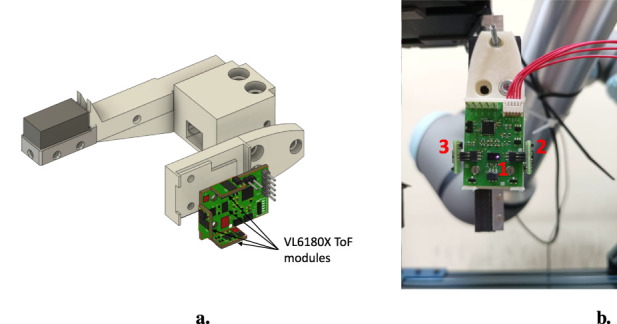
**(a)** CAD model of the proximity sensor. **(b)** Real proximity sensor with 3 ToF modules.

Figure 1a contains another part, i.e., the central support, which has not been mentioned previously. This component makes the end effector modular. In fact, it can be mounted on parallel grippers, and different tools can be attached to its sides. For example, Figure 1a shows two tactile fingers attached to the support, while in Figure 2a there is a tactile finger at one side and a proximity sensor at the other. Given the possibility to easily 3D print mechanical adapter, it is possible to create different configurations depending on the application requirements.

### 2.2 Proximity sensor

This section describes the proximity sensor, which is almost the same as the one reported in Cirillo et al. (2021b). However, in order to make the article self-contained, some details are reported here.

#### 2.2.1 Sensing technology

The developed sensor is a self-consistent board with a dedicated processing unit, capable of hosting up to 4 Time-of-Flight (ToF) modules, one of which is directly welded on the PCB. The CAD model of the sensor is shown in Figure 2a. The ToFs are based on the VL6180X chip (manufactured by STMicroelectronics) and, apart from the one welded on the main PCB, are embedded in Plug&Play modules whose dimensions are 12 mm ×8 mm and contain all the required electronics and a 6-way connector (manufactured by Samtec). The main PCB measures 24 mm ×34 mm and hosts a microcontroller (PIC16F19176 by Microchip) that communicates with the ToF modules through ${\text{I}}^{2}$ C interface and can be interrogated from an external device via serial interface. The main characteristics of the proximity sensor, such as sampling frequency, hysteresis, repeatability, and measurement errors, have been experimentally evaluated as detailed in (Cirillo et al., 2021b) and are reported in Table 2. Regarding the sampling frequency, it depends on the convergence time of the measurement which, in turn, depends on the obstacle distance. For this reason, the sampling frequency is fixed from the interrogating device. The latter is a computer where a ROS node communicates with the sensor at a frequency of 50 Hz. The values received by the sensor are the measured distances in mm. In particular, the same ROS node reported in the previous section is used to interrogate the proximity sensor too.

**TABLE 2:** Characteristics of the proximity sensor.

Maximum number of modules	4
Board dimensions	24×34 mm
Sampling frequency	[54.8,121.5] Hz
Hysteresis error	1.53%
Repeatability error	16% (at 10 mm) 13% (at 100 mm)
Measurement error	10%

#### 2.2.2 Mechanical design

A mechanical adapter to connect the proximity sensor to the central support has been designed and is shown in Figure 2a. This component is such that it can be fixed to the central support by means of two screws and can securely host the proximity board. Figure 2b shows a real sensor with three ToF modules: one is welded to the board (1) and two are connected through the available connectors, on the right (2) and on the left (3).

### 2.3 Human-robot interaction methodology

The objective is to demonstrate how it is possible to exploit the tactile and proximity sensors to allow human-robot interaction. Only as an example, in the considered application the tactile sensor is used to teach the robot the routing path for a wire, and the proximity sensor to avoid an obstacle or to follow the hand of an operator.

#### 2.3.1 Teaching by demonstration through tactile data

**FIGURE 3:**
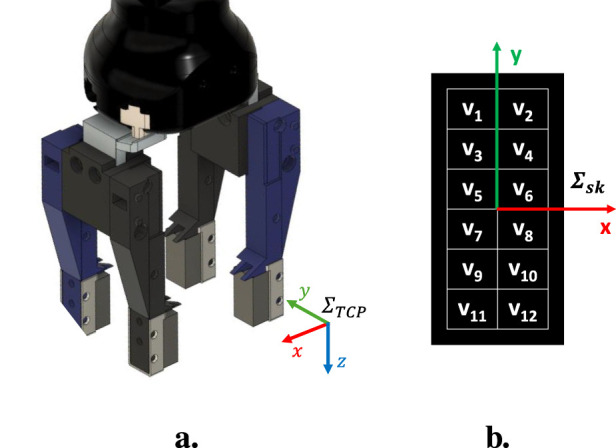
**(a)** Gripper with two pairs of tactile fingers (blue = active; black = passive) and orientation of TCP reference frame. **(b)** Voltage signals naming convention and reference frame $\left(\Sigma_{sk}\right)$ .

The configuration of the modular tools for this use case is shown in Figure 3a, where the orientation of the reference frame related to the Tool Center Point is also shown (its origin is at the center of the four fingers). It consists of two pairs of tactile fingers and each pairs has an active finger, i.e., with the tactile sensor inside, and a passive finger, i.e., without the sensing components. It is worth mentioning that the only reason for not having all active fingers is that at the time of writing only two PCBs with tactile components are available. However, the following can be easily extended to a setup with four active tactile fingers. The two tactile sensors are exploited to compute tactile indicators that are used to move the robot according to the direction of the force/torque exerted by an operator on the grasped object (an electrical wire in the considered task) during the teaching phase. In fact, thanks to the presence of a grid of taxels and the asymmetry of the optoelectronic component constituting each taxel (see Laudante et al., 2023), it is possible to detect deformations due to the application of shear forces on the deformable pad by tracking the displacement of the centroid of the tactile map. The tactile indicators are derived from the one defined in Caccavale et al. (2023) for a single tactile sensor, which has been extended to combine data from different sensors. For each of the two tactile sensors, the quantity $I_{ck}=\left(I_{xk},I_{yk}\right)$ , with k=1,2 indicating the corresponding tactile sensor, has two components $I_{xk}$ and $I_{yk}$ and represents the displacement of the centroid of the tactile map caused by external contact after grasping the object. These are computed as reported in Equation 1:

$$I_{xk}=x_{ck}-x_{\mathrm{ck0}},\;\;I_{yk}=y_{ck}-y_{\mathrm{ck0}},$$ (1)

where ( $x_{ck}$ , $y_{ck}$ ) and ( $x_{\mathrm{ck0}}$ , $y_{\mathrm{ck0}}$ ) are the coordinates of the tactile map centroid during the guiding operation and right after the grasp operation, respectively. In both cases, the coordinates are computed from the voltage signals $v_{ik}$ as reported in Equation 2:

$$x_{ck}=\frac{\sum_{i=1}^{12}v_{ik}x_{ik}}{\sum_{i=1}^{12}v_{ik}},y_{ck}=\frac{\sum_{i=1}^{12}v_{ik}y_{ik}}{\sum_{i=1}^{12}v_{ik}},$$ (2)

where ( $x_{ik}$ , $y_{ik}$ ) are the i−th taxel coordinates (expressed in mm) with respect to the $\Sigma_{sk}$ reference frame reported in Figure 3b. The indicator values computed in this way result expressed in mm.

The use of these indicators allows us to have a total of four independent components coming from the two tactile sensors which are related to the force and torque that an operator applies to the grasped object. In the case where the grasped object is a thin electrical wire, it is possible to distinguish up to four different movements by suitably combining $I_{x1}$ with $I_{x2}$ and $I_{y1}$ with $I_{y2}$ , as discussed in the following.

**FIGURE 4:**
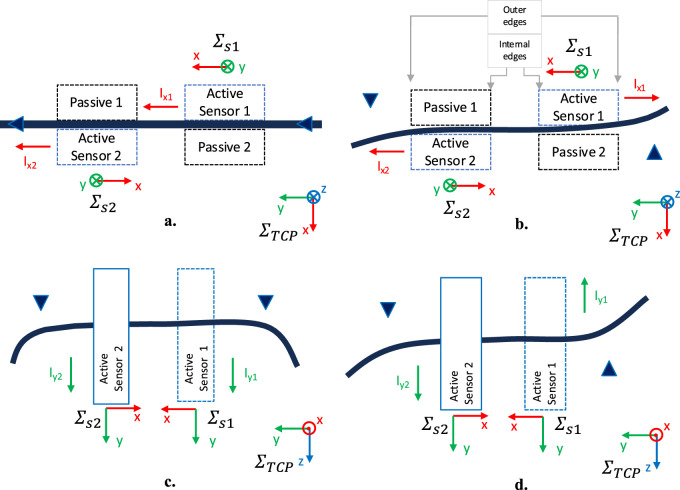
Schematic representations of the contact indicator behaviour in different conditions: **(a)** force applied along y -axis of the $\Sigma_{\mathrm{TCP}}$ (top view); **(b)** moment applied about z -axis of $\Sigma_{\mathrm{TCP}}$ (top view); **(c)** force applied along z -axis of $\Sigma_{\mathrm{TCP}}$ (frontal view); **(d)** moment applied about x -axis of $\Sigma_{\mathrm{TCP}}$ (frontal view).

The movement of the grasped object along the y -axis of $\Sigma_{\mathrm{TCP}}$ shown in Figure 3a affects only the $I_{xk}$ component of the indicators. In this case, if a force is applied on the object along the positive y -direction of the Tool Center Point (TCP) $\Sigma_{\mathrm{TCP}}$ frame, the centroid of the tactile map of both sensors moves in the same direction. In the schematized example in Figure 4a, $I_{x1}$ becomes positive with respect to $\Sigma_{s1}$ and $I_{x2}$ negative with respect to $\Sigma_{s2}$ , vice versa if the object is pulled along the negative y -direction.

Instead, considering the example schematized in Figure 4b where a torque is applied about the z -axis of $\Sigma_{\mathrm{TCP}}$ , $I_{x1}$ and $I_{x2}$ have the same sign in the respective frames $\Sigma_{s1}$ and $\Sigma_{s2}$ , which is positive if the rotation is counterclockwise and negative if it is clockwise. This happens because the grasped object tends to push on the outer edges of both sensors, releasing the inner edges. The opposite occurs when the rotation is counterclockwise. To correctly distinguish the two cases, i.e., torque about z -axis and force along y -direction, the $I_{x1}$ and $I_{x2}$ components are combined, by introducing the two interaction indicators $I_{\mathrm{transly}}$ for translations and $I_{\mathrm{rotz}}$ for rotations, defined as follows:

$$I_{\mathrm{transly}}=\frac{I_{x1}-I_{x2}}{2},$$ (3)

$$I_{\mathrm{rotz}}=\frac{I_{x1}+I_{x2}}{2}.$$ (4)

The value of the $I_{\mathrm{transly}}$ indicator is positive (negative) if the grasped object is pulled along the positive (negative) y -direction, while its value is approximately zero if the object is purely rotated about the z axis. In contrast, the value of the indicator $I_{\mathrm{rotz}}$ is approximately zero if the object is pulled along the y -direction, while it is positive (negative) in the case of a counterclockwise (clockwise) rotation about the z -axis. Obviously, simultaneous forces and torques can be managed by considering the two indicators at the same time.

When a force is exerted on the object along the positive z -direction of the frame $\Sigma_{\mathrm{TCP}}$ as depicted in Figure 4c, the values of the components $I_{yk}$ are different from zero. In particular, $I_{y1}$ and $I_{y2}$ are both positive with respect to the corresponding sensor frames $\Sigma_{s1}$ and $\Sigma_{s2}$ if the object moves along the z -positive direction and both negative in the opposit case. Instead, if a torque is applied about the x -axis of $\Sigma_{\mathrm{TCP}}$ as reported in Figure 4d, the values of the components $I_{yk}$ are opposite in sign. In detail, the value of $I_{y1}$ is negative (positive) and the value of $I_{y2}$ is positive (negative) when the object is rotated counterclockwise (clockwise). To correctly discriminate these two cases, where the $I_{yk}$ components are involved, the following two interaction indicators are introduced:

$$I_{\mathrm{translz}}=\frac{I_{y1}+I_{y2}}{2},$$ (5)

$$I_{\mathrm{rotx}}=\frac{I_{y2}-I_{y1}}{2}.$$ (6)

The $I_{\mathrm{translz}}$ indicator is positive (negative) if a force along the positive (negative) z -direction of $\Sigma_{\mathrm{TCP}}$ is applied on the object, while it is approximately zero if the object is subjected to a pure rotation about the x -axis of $\Sigma_{\mathrm{TCP}}$ . The value of $I_{\mathrm{rotx}}$ , instead, is approximately zero if the object is pulled along the z -direction of $\Sigma_{\mathrm{TCP}}$ , while it is positive (negative) in case of a counterclockwise (clockwise) rotation about the x -axis. Once again, combined motions comprising both forces along z and torques about x can be managed by looking at the two defined indicators at the same time.

To accomplish the hand guidance phase, velocity commands for the $\Sigma_{\mathrm{TCP}}$ frame are directly computed from the interaction indicators in Equations 3–6. First, a saturated dead-zone function is applied to all indicators. The dead-zone is used to fix the velocity to zero when the indicators assume low values, especially due to signal noise. The saturation is exploited to limit the maximum interaction velocity. The slope can be selected to increase or decrease the response of the robot’s velocity to the operator’s actions based on the task needs. In practice, the slope represents the indicator-velocity gain used to guide the robot. For each indicator, a different saturated dead-zone function can be defined. In detail, four dead-zone functions have to be selected: the first to compute the linear velocity $u_{y}$ of the $\Sigma_{\mathrm{TCP}}$ frame in the y direction from the value of $I_{\mathrm{transly}}$ indicator; the second to compute the angular velocity $\omega_{z}$ about the z axis of the $\Sigma_{\mathrm{TCP}}$ frame from the value of $I_{\mathrm{rotz}}$ indicator; the third to compute the linear velocity $u_{z}$ of the $\Sigma_{\mathrm{TCP}}$ frame in the z direction from $I_{\mathrm{translz}}$ value; the last one to compute the angular velocity $\omega_{x}$ about the x axis of the $\Sigma_{\mathrm{TCP}}$ frame from $I_{\mathrm{rotx}}$ value.

#### 2.3.2 Interaction through proximity sensor data

**FIGURE 5:**
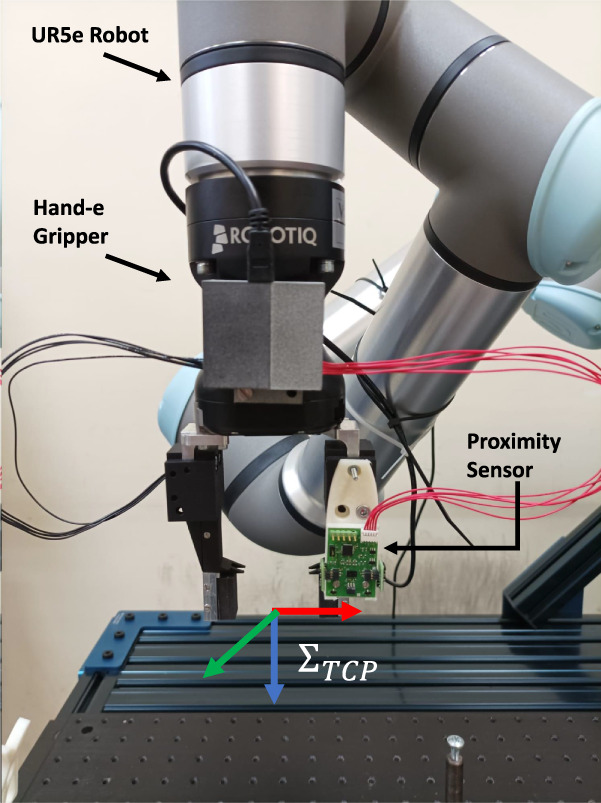
Experimental setup with $\Sigma_{\mathrm{TCP}}$ frame.

The proximity sensor board can be exploited to accomplish, for example, *collision avoidance* or *user-following* tasks. In collision avoidance mode, the data acquired from the proximity sensors are used to detect an obstacle, e.g., a human operator working in the cell, so that the robot arm can retract and avoid collision. In particular, fixed a safety distance $d_{j}^{\mathrm{limit}}$ for each ToF module, when the distance $d_{j}$ measured by the module is less than the safety distance, a correction is applied to the movement the robot is executing. The proposed correction is a variation on the robot linear velocity along the corresponding direction of $\Sigma_{\mathrm{TCP}}$ (see Figure 5), and it is computed as in Equation 7:

$$\left\{\begin{array}{cc} u=g\cdot \left(d_{j}-d_{j}^{\mathrm{limit}}\right),\; & \text{if }d_{j}\leq d_{j}^{\mathrm{limit}}\\ u=0,\; & \text{if }d_{j}>d_{j}^{\mathrm{limit}} \end{array}\right.$$ (7)

where g is a gain that can be selected in order to tune system sensitivity.

In user-following mode, the objective is to control the linear velocity of the robot to keep the measured distance equal to a desired value $d_{j}^{\mathrm{des}}$ . In this modality, it is possible, for example, to move the robot arm or teach it a simple trajectory in a contactless manner. The implementation can be done by computing the velocities with respect to $\Sigma_{\mathrm{TCP}}$ frame as in Equation 8:

$$u=g\cdot \left(d_{j}-d_{j}^{\mathrm{des}}\right).$$ (8)

The parameter $d_{j}^{\mathrm{limit}}$ is used to start and stop the user-following mode. For instance, the robot starts to follow the object, e.g., the operator’s hand, when it comes to a distance less than $d_{j}^{\mathrm{limit}}$ and stops to follow it when it is removed, i.e., the measured distance becomes greater than $d_{j}^{\mathrm{limit}}$ . Resuming, the interaction with proximity sensors proposed in this work can be summarized with the pseudo-code Algorithm 1.

Algorithm 1 Interaction with proximity sensors.

**procedure** Interaction Proximity
** if** collision_avoidance_mode **then**
**  if** $d_{j}\leq d_{j}^{\mathrm{limit}}$ **then**
**   **

$u\leftarrow g\cdot \left(d_{j}-d_{j}^{\mathrm{limit}}\right)$

**  else**
**   **

u←0

**  end if**
** end if**
** if** user_following_mode **then**
**  if** $d_{j}\leq d_{j}^{\mathrm{limit}}$ **then**
**   **

$u\leftarrow g\cdot \left(d_{j}-d_{j}^{\mathrm{des}}\right)$

**  else**
**   **

u←0

**  end if**
** end if**
**end procedure**

## 3 Results

A series of demonstrations have been carried out to validate the proposed methodology. The setup is constituted by a Universal Robot UR5e manipulator equipped with a Robotiq Hand-E gripper on which the modular sensing system has been mounted. The first scenario aims to demonstrate the potential of the teaching-by-demonstration methodology during the manipulation of a wire. The second and third ones show the possibilities of human-robot interaction using the proximity sensor for collision avoidance and user-following tasks. The last use case combines the use of tactile and proximity sensors to guide the robot while avoiding obstacles.

### 3.1 Teaching by demonstration

**FIGURE 6:**
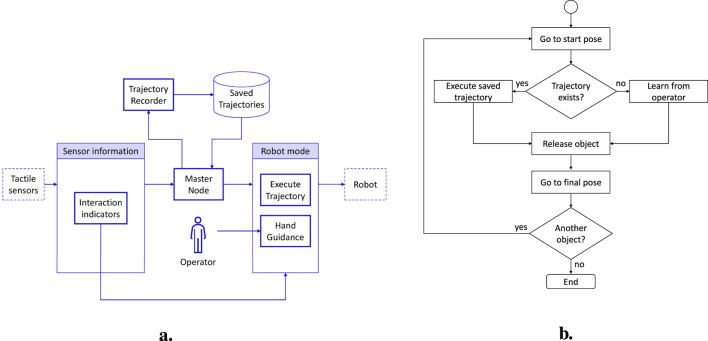
**(a)** Software architecture for the teaching-by-demonstration use case. **(b)** Flowchart of the teaching-by-demonstration task.

The considered task consists in teaching the robot the path to follow for routing a wire. From the starting point where the wire is grasped, the operator guides the robot by exploiting the proposed indicators during the interaction with the grasped wire. At the end of the routing path, the wire is inserted into a clip. The trajectory followed during the teaching phase is saved so that it can be then re-executed autonomously by the robot. Task management requires a software architecture (reported in Figure 6a) with a ROS node that orchestrates the overall execution. In particular, after the wire grasping, the master node checks if a trajectory is available. In case a trajectory exists, it is autonomously executed; otherwise the hand guidance mode starts. The flowchart in Figure 6b summarizes the task operations. Additionally, during autonomous trajectory execution, it is possible to check if the wire gets entangled by monitoring the same indicators used during the teaching. In particular, the robot motion is stopped if the indicator $I_{\mathrm{transly}}$ reaches a fixed threshold value.

As already discussed, the saturated dead-zone functions have to be tuned before computing velocities from indicators. In detail, the following parameters have been chosen for the realized application:

1. for $I_{\mathrm{transly}}$ indicator: saturation value equal to 0.08 m/s , slope equal to 0.40 m/(mm⋅s) , dead-zone equal to 0.08 mm

2. for $I_{\mathrm{rotz}}$ indicator: saturation value equal to 0.20 rad/s , slope equal to 0.70 rad/(mm⋅s) , dead-zone equal to 0.08 mm

3. for $I_{\mathrm{translz}}$ indicator: saturation value equal to 0.08 m/s , slope equal to 0.08 m/(mm⋅s) , dead-zone equal to 0.2 mm

4. for $I_{\mathrm{rotx}}$ indicator: saturation value equal to 0.20 rad/s , slope equal to 0.10 rad/(mm⋅s) , dead-zone equal to 0.50 mm

As said, it is possible to adapt the function parameters to the experiments by taking into account the characteristics of the sensor used, e.g., signal noise and sensitivity which are typically sensor dependent. In our case, for example, the values reported above take into account that the sensitivity of the indicator in the y -direction is greater than that in the x -direction.

**FIGURE 7:**
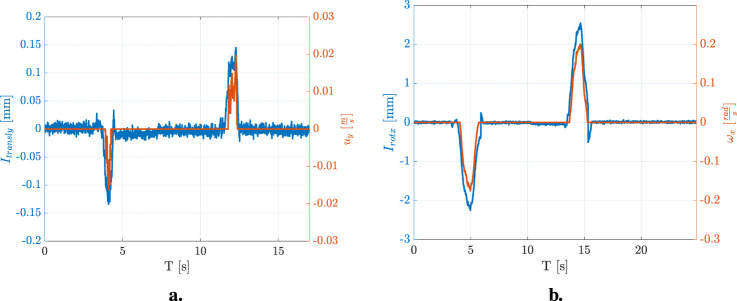
**(a)** Indicator $I_{\mathrm{transly}}$ and commanded velocity $u_{y}$ . **(b)** Indicator $I_{\mathrm{rotx}}$ and commanded velocity $\omega_{x}$ .

In order to show the effects of the chosen indicator-velocity functions, two examples (one for the linear velocity and one for the angular velocity) are reported. Figure 7a reports the indicator $I_{\mathrm{transly}}$ and the corresponding linear velocity $u_{y}$ when a force on the grasped object is first applied in the negative y -direction of $\Sigma_{\mathrm{TCP}}$ and then in the positive y -direction. Figure 7b, instead, shows the indicator $I_{\mathrm{rotx}}$ and the corresponding angular velocity $\omega_{x}$ obtained when a torque is applied about the x -axis of $\Sigma_{\mathrm{TCP}}$ .

**FIGURE 8:**
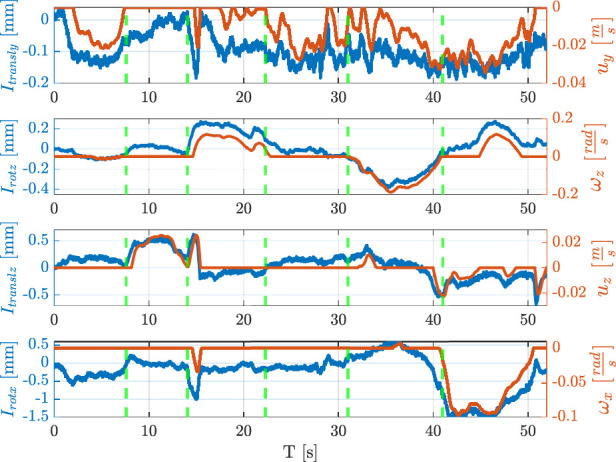
Indicators and corresponding velocities during the teaching phase.

**FIGURE 9:**
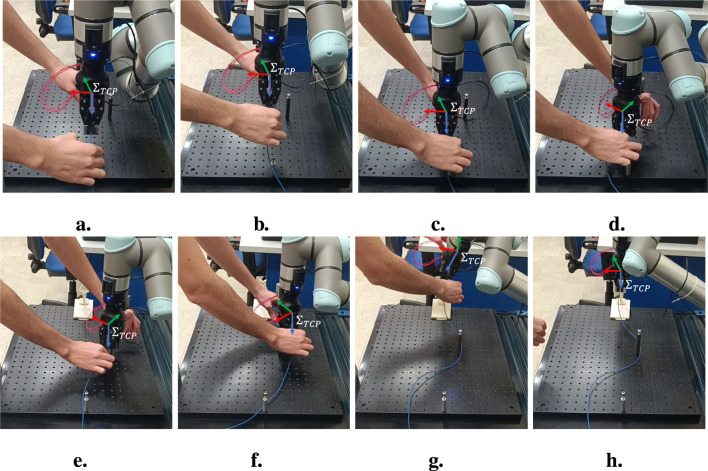
Operator guiding the robot by acting on the grasped electrical wire. From the starting point **(a)**, the operator moves the robot along the y -axis (in green) by pulling the wire in the corresponding direction **(b)**. The robot is then moved downward by pushing the wire down **(c)** and rotated anti-clockwise about the z -axis (in blue) **(d)**. The robot is then translated again along the y -axis and rotated in the opposite direction **(e, f)**. Finally, the robot end effector is translated and rotated to be positioned above the target clip **(g)**, where the wire is then inserted **(h)**.

**FIGURE 10:**
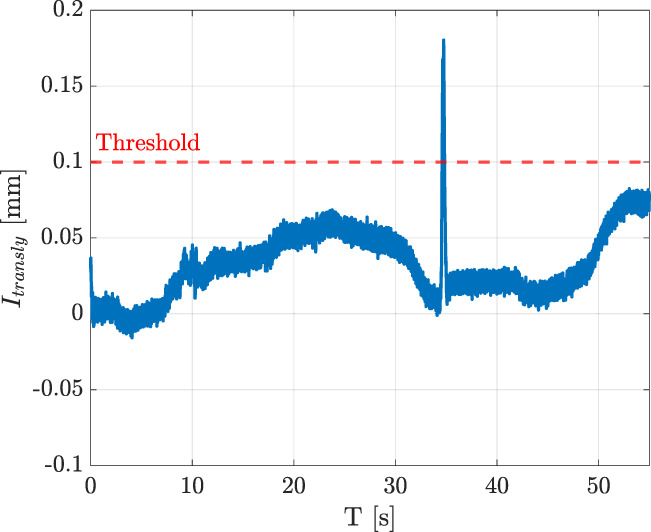
Indicator $I_{\mathrm{transly}}$ during the autonomous wire routing.

The whole routing task has been executed as described in the following. At the beginning no trajectory is saved into the database, so the robot is set in hand-guidance mode. Hence, the operator guides the robot through the desired path by exerting forces or torques on the wire grasped by the modular sensors. Figure 8 shows the value of the indicators and the corresponding velocities during the whole experiment, which can be divided into six intervals. From the starting point (Figure 9a), the robot is moved in the y -direction (time interval [0,8] s, see Figure 9b), then it is translated in the z -direction (interval [8,14] s, see Figure 9c). Next, the $\Sigma_{\mathrm{TCP}}$ frame is rotated about the z -axis (interval [14,22] s, see Figure 9d), then a translation in the y -direction is applied (interval [22,31] s, see Figure 9e), and the $\Sigma_{\mathrm{TCP}}$ frame is rotated about z in the opposite direction (interval [31,41] s, see Figure 9f). Finally, the robot is positioned above the clip applying simultaneous rotations about the x and the z -axes, and a translation in the y -direction (see Figure 9g). Finally, the robot inserts the wire into the clip (see Figure 9h). After the teaching phase, the robot autonomously executes the wire routing task. In this phase, possible wire entanglements are evaluated by comparing the indicator $I_{\mathrm{transly}}$ with a fixed threshold equal to 0.10 mm. Figure 10 reports the indicator $I_{\mathrm{transly}}$ during the autonomous wire routing. While executing the task, an entanglement is simulated on purpose at the instant t=34 s, and the robot consequently stops. Then, the operator frees the wire and the routing operation is resumed. The threshold can be decreased or increased to have more or less sensitivity, respectively. The video attached to the paper shows the described experiment in detail.

### 3.2 Collision avoidance and user-following

**FIGURE 11:**
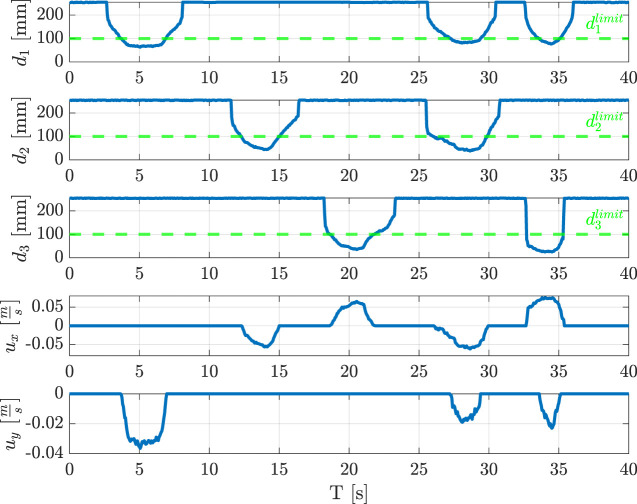
Measured distance and commanded velocity during the collision avoidance experiment.

**FIGURE 12:**
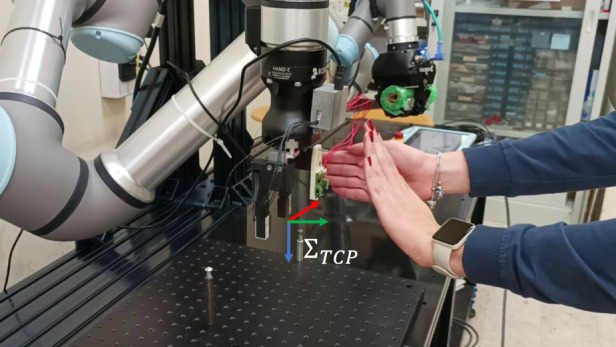
Collision avoidance involving two proximity sensor modules. The operator simulates obstacles on two sides of the end effector and the robot reacts by moving away from the operator’s hands.

A demonstration with an operator who approaches the gripper with his hand has been implemented to show the potential of the proposed collision avoidance methodology. Figure 11 reports the distance data acquired from the three proximity sensor modules and the corresponding velocities with respect to $\Sigma_{\mathrm{TCP}}$ frame. The test is composed of five interactions: the first three involve one sensor module at a time, modules #1 , #2 and #3 are approached in order, while the last two use the combination of two sensor modules, i.e., #1 and #2 first, and #1 and #3 later. In the first three cases, only one velocity component changes when the distance $d_{j}$ becomes less than the limit value, i.e., $d_{j}^{\mathrm{limit}}=100$ mm. In particular, the first case causes the velocity component $u_{y}$ to go from zero to a negative value. Instead, the second and third cases affect the velocity component $u_{x}$ with opposite signs, since the human hand moves towards the modules from opposite directions. Subsequently, the operator’s hands get closer to both sensor modules #1 and #2 (see Figure 12), and finally to both sensor modules #1 and #3 . As a consequence, the robot moves along both x and y axes, with the proper signs of the velocity components.

**FIGURE 13:**
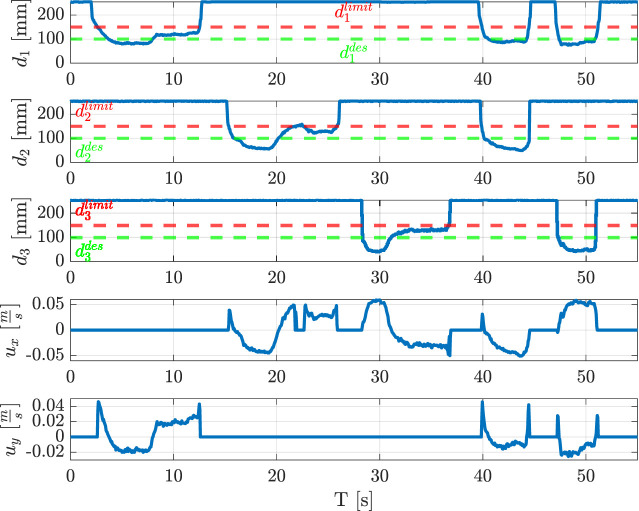
Distance measured and commanded velocity during the user following experiment.

A similar test has been carried out to show the capability of the user-following modality. As for the previous experiment, Figure 13 shows the distances $d_{j}$ measured by the proximity sensors and the corresponding velocities. The parameters used in the tests are set to 150 mm for the limit distance $d_{j}^{\mathrm{limit}}$ and to 100 mm for the desired distance $d_{j}^{\mathrm{des}}$ . The sensor modules have been tested in the same order and with the same combinations as in the experiments for collision avoidance. Similarly, the robot moves along only one direction during the first three events, the y direction in the first case and the x direction in the second and the third ones. The robot follows the human hand, which first approaches the sensor and then moves away from it. Then, the remaining two cases are characterized by the combined use of two modules, so when the hands come near the two sensors, the robot shows velocities in both the x and y directions. The video attached to the paper shows also these demos in detail.

### 3.3 Collision avoidance during hand guidance

**FIGURE 14:**
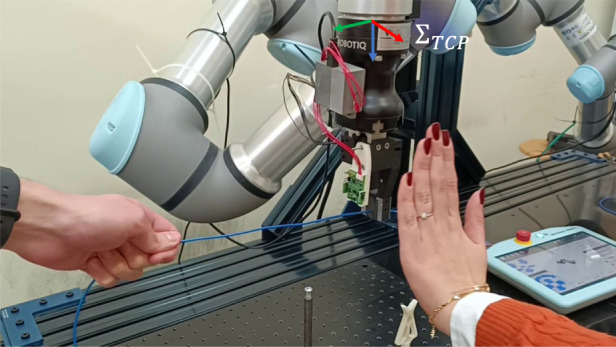
Hand guidance and obstacle avoidance. An operator guides the robot by pulling the grasped wire while another operator simulates an obstacle nearby the end effector. The robot reacts by following the operator guidance but moving far from the obstacle.

**FIGURE 15:**
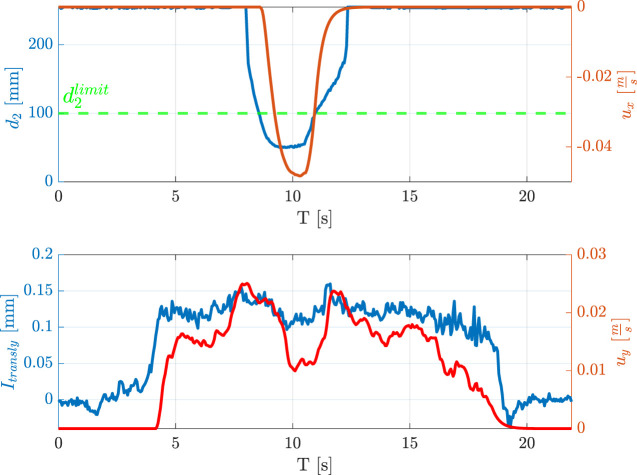
Measured distance, indicator, and velocities during the obstacle avoidance in hand guidance experiment.

The last test intends to demonstrate how it is possible to combine the use of the interaction indicators computed from tactile data with the proximity sensor data, implementing more complex tasks. In detail, an operator can interact with the robot through the grasped wire and, at the same time, the robot can avoid collisions. In this case, the modular sensors mounted on the gripper are constituted by a pair of fingers for grasping the wire, and a proximity sensor. The wire is pulled along the y direction of $\Sigma_{\mathrm{TCP}}$ from a distance greater than the previous cases and, while the robot follows this direction by exploiting indicator $I_{\mathrm{transly}}$ , another operator, i.e., an obstacle, approaches it from the x direction, by activating the collision avoidance for the sensor module #2 (see Figure 14). Figure 15 shows the distance measured by the proximity module, the indicator $I_{\mathrm{transly}}$ , and the computed $u_{x}$ and $u_{y}$ velocities used for the robot control. As for the previous ones, also this demo is reported in the video attached to the paper.

## 4 Discussion

This paper proposed a robotic system, considering both hardware and software technologies, that can be used in those tasks where human-robot interaction is requested. Regarding hardware, a modular system that can be mounted on commercial parallel grippers has been developed, and, for the tasks considered, it has been equipped with tactile and/or proximity sensors. Differently from previous works that proposed systems integrating tactile and proximity sensors for safe HRI applications, the proposed hardware is such that it can be directly mounted on the robot’s parallel gripper and does not require additional tools/skins on/near the end effector. In addition to the hardware, the methodology for using the aforementioned sensors has been detailed. For instance, tactile sensors have been exploited as a human-robot interface to implement a teaching-by-demonstration application in which an operator guides a robotic arm through the routing path for an electrical cable. Finally, proximity sensors have been used both as a safety system, implementing a collision avoidance mechanism, and as an interface for moving the robot end effector in a contactless fashion. Demonstrations for all the proposed algorithms have been presented, showing their effectiveness. While the use cases showcased in this paper only consider the manipulation of electrical wires, we expect that the algorithms proposed here can be used with objects of different shapes. This aspect will be evaluated in future studies. In addition, with regard to the modularity of the developed system, there are several possible future developments. In fact, by changing the sensors or tools on the end effector, the system can be adapted to the specific application. One example could be the integration of cameras and the implementation of computer vision algorithms to recognize and locate the object(s) of interest in the scene, providing even more autonomy to the robot when needed.

## Data Availability

The raw data supporting the conclusions of this article will be made available by the authors upon request, without undue reservation.
